# Matrix Metalloproteinase Polymorphisms as Genetic Risk Factors for Anterior Cruciate Ligament Injuries in Football Players: A Case–Control Study

**DOI:** 10.3390/genes16121505

**Published:** 2025-12-16

**Authors:** Kinga Wiktoria Łosińska, Agata Rzeszutko-Bełzowska, Krzysztof Ficek, Myosotis Massidda, Giovanna Maria Ghiani, Paweł Cięszczyk, Alison Victoria September

**Affiliations:** 1 Department of Molecular Biology, Gdansk University of Physical Education and Sport, Kazimierza Górskiego 1, 80-336 Gdansk, Poland; 2 Faculty of Physical Culture Sciences, University of Rzeszów Cicha 2a, 35-326 Rzeszów, Poland; arzeszutko@ur.edu.pl; 3 Department of Physiotherapy in Musculoskeletal Dysfunctions, Jerzy Kukuczka Academy of Physical Education in Katowice, Mikolowska 72a, 40-065 Katowice, Poland; k.ficek@awf.katowice.pl; 4 Department of Medical Sciences and Public Health, University of Cagliari, 09124 Cagliari, Italy; myosotis.massidda@unica.it; 5 Sports Physiology Laboratory, University of Cagliari, Università 40, 09124 Cagliari, Italy; giovannam.ghiani@unica.it; 6 Health Through Physical Activity, Lifestyle and Sport Research Centre (HPALS), Division of Physiological Sciences, Department of Human Biology, University of Cape Town, Rondebosch, Cape Town 7700, South Africa; alison.september@uct.ac.za

**Keywords:** ACL injury, sports medicine, case-control study, matrix metalloproteinases (MMPs)

## Abstract

**Background/Objectives**: Injuries to the anterior cruciate ligament (ACL) frequently occur in physically active populations and often lead to long-term complications, such as osteoarthritis and recurrent injury. The ACL’s structural integrity depends on extracellular matrix (ECM) remodeling, regulated by matrix metalloproteinases (MMPs). This study examined the association between three polymorphisms—MMP1 rs1799750, MMP10 rs486055, and MMP12 rs2276109—and ACL injury outcomes, including injury frequency, strain, partial rupture, and complete rupture. **Methods**: A total of 296 physically active, unrelated Caucasian males participated in this case–control study, including 160 with ACL injuries (classified as ACLF—ACL injury frequency, ACLS—strain, ACLRP—partial rupture, ACLRC—complete rupture, and ACL—general ACL injury) and 136 healthy controls (CON) with no previous ACL injuries. All injuries resulted from non-contact mechanisms. **Results**: The MMP1 rs1799750 polymorphism showed a protective effect against ACL injury compared to controls (OR = 0.42, 95% CI: 0.21–0.85, Padj = 0.014). Within the injury group, MMP10 rs486055 was significantly associated with partial ruptures, especially in heterozygous carriers (OR = 3.47, 95% CI: 1.64–7.33, *p* = 0.001). The MMP12 rs2276109 variant, under a dominant model, was linked to higher injury frequency (OR = 3.80, 95% CI: 1.69–8.54, *p* = 0.0009) but showed no association with injury severity. **Conclusions**: The *MMP1* rs1799750 polymorphism showed a protective effect against ACL injury, *MMP10* rs486055 was associated with an increased risk of partial rupture, and *MMP12* rs2276109 was linked to higher injury frequency. These findings highlight the complex genetic and biomechanical interactions underlying ACL injuries. The *MMP1* rs1799750 polymorphism showed a protective effect (58% reduction in the odds compared to controls) against ACL injury, *MMP10* rs486055 was associated with an increased risk (3.47 times higher odds) of partial rupture, and *MMP12* rs2276109 was linked to 3.8 times higher odds of an injury. Identifying genetic risk factors may support personalized injury prevention and rehabilitation strategies, offering new opportunities to reduce long-term complications in athletes and active individuals.

## 1. Introduction

Anterior cruciate ligament (ACL) injuries are among the most prevalent and debilitating musculoskeletal injuries, particularly in athletes and physically active individuals. These injuries not only disrupt athletic careers and daily functioning but also pose a significant economic and healthcare burden. ACL injuries are associated with high rates of recurrence and long-term complications, such as osteoarthritis, with nearly 50% of patients developing post-traumatic osteoarthritis within a decade of injury. Despite advances in surgical techniques and rehabilitation protocols, these complications emphasize the need for a deeper understanding of the factors that predispose individuals to ACL injuries and affect recovery outcomes [1,2,3].

The structural integrity and functional resilience of the ACL are primarily determined by the extracellular matrix (ECM), a complex and highly organized network of proteins, such as collagens, glycoproteins, and proteoglycans. This matrix not only provides tensile strength and load distribution to the ligament but also mediates its response to mechanical stress and injury. Matrix metalloproteinases (MMPs), a family of zinc-dependent proteases, are critical regulators of ECM remodeling. MMPs degrade and recycle ECM components, such as collagens and glycoproteins, and play key roles in tissue adaptation, injury repair, and inflammation [4,5]. However, dysregulation of MMP activity can lead to excessive ECM degradation, impairing tissue integrity and increasing susceptibility to ligament injuries [6].

Genetic factors are increasingly recognized as important contributors to ACL injury risk. Heritability studies estimate that genetic factors account for up to 69% of the predisposition to ACL injuries [7,8,9]. Variants in genes which encode for the structural components of the ECM, such as collagen type I alpha 1 (COL1A1), collagen type V alpha 1 (COL5A1), and tenascin C (TNC), have been associated with ligament pathology and injury risk [2,10,11]. ECM regulators, such as matrix metalloproteinases (MMPs), have also emerged as strong candidates for genetic association studies. Variants in MMP genes, including MMP1, MMP3, and MMP12, have been linked to musculoskeletal injuries, such as Achilles tendinopathy and ACL ruptures [12]. These may modulate MMP expression and activity, potentially influencing ligament remodeling, resilience under mechanical stress, and susceptibility to rupture [13,14].

Among the most studied MMPs, MMP1 rs1799750, MMP10 rs486055, and MMP12 rs2276109 have been implicated in ligament injuries and related conditions. MMP1 rs1799750, located in the gene’s promoter region, affects transcriptional activity and may alter collagen degradation and ligament repair dynamics [15,16]. MMP10 rs486055, a non-synonymous variant, influences stromelysin activity, potentially affecting ECM remodeling during partial ruptures [17]. MMP12 rs2276109, another promoter variant, modulates the gene’s expression and has been associated with injury recurrence, particularly in the context of ligament repair and chronic mechanical stress [17,18]. However, the specific contributions of these polymorphisms to ACL injuries remain poorly understood.

Environmental and biomechanical factors further interact with genetic predispositions to exacerbate injury risk. High-intensity sports and repetitive mechanical loading may amplify the effects of MMP dysregulation, particularly in individuals with high-risk genotypes [3,19]. Moreover, epigenetic modifications, such as DNA methylation and histone acetylation, can regulate MMP expression in response to environmental stressors, adding another layer of complexity to injury susceptibility [20,21]. Exploring how these factors interact is critical for developing predictive models and targeted interventions for ACL injury prevention.

For these reasons, the study aimed to investigate genetic polymorphims within key *MMP* genes, namely *MMP1* rs1799750, *MMP10* rs486055, and *MMP12* rs227610, and the risk of ACL-related injuries, including injury frequency, strain, partial rupture, and complete rupture. By employing various genetic models, we sought to elucidate the role of these polymorphisms in ACL pathophysiology. This research builds on previous studies by integrating genetic and biomechanical insights to provide a more comprehensive understanding of ACL injuries. Ultimately, these findings could pave the way for personalized approaches to injury prevention, diagnosis, and rehabilitation. Therefore, the primary aims of this study were to determine whether *MMP1* rs1799750, *MMP10* rs486055, and *MMP12* rs2276109 polymorphisms were associated with either susceptibility to ACL injuries and if there was an association with the severity of the ACL injuries sustained in football players.

## 2. Materials and Methods

### 2.1. Participants

A total of 296 physically active, unrelated, self-identified Caucasian individuals were enrolled in this case–control genetic association study between 2017 and 2024. The participants included 160 males who had suffered ACL injuries (classified as ACLS—strain, ACLRP—partial rupture, and ACLRC—complete rupture) and 136 healthy male controls with no prior history of ACL injuries (CON group). The classification of ACL injuries (strain, partial rupture, and complete rupture) was determined based on clinical examination and confirmed by magnetic resonance imaging (MRI) or ultrasound analyses performed by an experienced sports medicine specialist. All ACL injuries in the study were sustained through non-contact mechanisms.

The injured participants were soccer players competing in the Polish 1st, 2nd, and 3rd division leagues, training between 11 and 14 h per week. The control group primarily consisted of soccer players who self-reported no history of ligament or tendon injuries. All male participants, both the injured and control groups, belonged to the same soccer teams, were of the same ethnicity (self-reported Polish, Eastern European ancestry for at least three generations), and had similar training loads and exposure to ACL injury risk (identical training and match intensity and volume).

### 2.2. Ethics Committee

The study was admitted by the Gdańsk University of Physical Education Ethics Committee (Uchwała nr 1/2024), and written informed consent was acquired from each participant according to the declaration of Helsinki [22].

### 2.3. Genetic Analyses

Genomic DNA was isolated from buccal swab samples using the GenElute Mammalian Genomic DNA Miniprep Kit (Sigma, Steinheim, Germany), following the manufacturer’s protocol. Genotyping was performed in duplicate with TaqMan^®^ Pre-Designed SNP Genotyping Assays (Applied Biosystems, Waltham, MA, USA) on a CFX96 Touch Real-Time PCR Detection System (BIO-RAD, Feldkirchen, Germany), according to the provided guidelines. To identify the MMP variants rs1799750, rs486055, and rs2276109 we used allelic discrimination assays with the following ID codes: C__34384693_10, C____632734_30, and C__15880589_10. The contained primers and fluorescently-labeled (FAM and VIC) probes were applied.

### 2.4. Statistical Analysis

The association between the *MMP1*: rs1799750 (NG_011740.2:g.3471del), *MMP10*: rs486055 (NM_002425.3:c.158G>C, NM_002425.3:c.158G>A), and *MMP12*: rs2276109 (NG_032936.1:g.4974A>T, NG_032936.1:g.4974A>G, NG_032936.1:g.4974A>C) and ACL injury status and injury-related outcomes within the case cohort was analyzed. The investigation was divided into two parts, namely a primary case–control comparison between injured and control groups and a secondary analysis focusing solely on injured participants. Within the ACL injury group, outcomes included ACLF, ACLS, ACLRP, and ACLRC. Both parts involved single-locus and haplotype-based analyses. Single-locus analysis was conducted using the R package SNPassoc (version 2.1-0) under four genetic models, namely codominant, dominant, recessive, overdominant. In all models, the minor allele was considered the risk factor. Odds ratios (ORs) with 95% confidence intervals (CIs) were calculated for each SNP across all genetic models, adjusting for potential confounders, specifically age and body mass.

We conducted a post hoc sensitivity analysis to calculate the minimal detectable odds ratio (MDOR) for case–control design for three genetic models. Power was computed with the genpwr.calc function (R, package genpwr), assuming a two-sided α = 0.05, the observed case/control ratio, and minor allele frequencies (MAFs) estimated in controls. For each SNP and genetic model (additive, dominant, or recessive), we searched over a grid of odds ratios to find the smallest OR achieving 80% and 90% power.

Haplotype-based analysis was conducted using the R haplo.stats package (version 1.9.7). Haplotypes were reconstructed via the expectation-maximization (EM) algorithm to account for phase ambiguity. Associations between haplotypes and ACL injury outcomes were assessed using a generalized linear model (GLM) approach (haplo.glm function), incorporating the uncertainty from haplotype estimation. The most frequent haplotype served as the reference for comparisons. As in single-locus analysis, adjustments were made for age and body mass.

For the multi-locus analysis, we used logic regression, implemented in the LogicFS R package (version 2.22.0). Each SNP variable, originally coded as 1 for homozygous references, 2 for heterozygous variants, and 3 for homozygous variants, was transformed into two binary dummy variables (SNP_1 and SNP_2) with the following encoding: genotype 1 was represented as SNP_1 = 0 and SNP_2 = 0; genotype 2 was represented as SNP_1 = 1 and SNP_2 = 0; and genotype 3 was represented as SNP_1 = 1 and SNP_2 = 1. The logic regression model was fit using the *logicFS* function with the default simulated annealing algorithm and 100 bootstrap, with up to 10 leaves per logic tree. The analysis was repeated 200 times—using the original dataset with true class labels and using a null dataset in which the class labels were randomly permuted. The importance of each interaction (i.e., the Boolean combination of SNP dummy variables) was assessed using the following variable importance measures: proportion-based importance, the proportion of logic trees in which a given interaction appeared, and the out-of-bag (OOB) importance, i.e., the average change in classification accuracy on OOB samples when a specific interaction was removed from the model. Only interactions with a mean proportion greater than 0.15 across the 200 iterations were considered. Bootstrap-based 95% confidence intervals were calculated for OOB importance for the real and the null dataset to allow statistical inference. As 3 SNPs (each represented by 2 binary variables) were included in the analysis, there were 64 possible genotype combinations across all loci. The logic regression algorithm searched this space to identify Boolean combinations of these binary predictors that best discriminate the outcome. The simulated annealing procedure used in LogicFS explored only those combinations that best discriminate the outcome.

Differences in demographic characteristics between groups were analyzed using the Wilcoxon test for continuous variables and Fisher’s exact test for categorical variables. A *p*-value less than 0.05 was considered statistically significant for all analyzes.

## 3. Results

Table 1 summarizes the characteristics of the case and control groups. In our primary case–control analysis, we examined the genotype distributions of the studied SNPs, comparing their frequencies between cases and controls. Given the notable demographic differences between the two groups—specifically in age (controls: 27 ± 4 years vs. cases: 36 ± 7 years, *p* < 0.001) and body mass (controls: 73 ± 16 kg vs. cases: 78 ± 10 kg, *p* < 0.001)—we adjusted the analysis accordingly. These adjustments were implemented to mitigate the potential confounding influence of age and body mass, which showed statistically significant disparities between the groups (as seen in Table 1). This approach was intended to better isolate and assess the independent genetic contribution of the SNPs to the observed differences between cases and controls.

MDORs at 80% power were 1.6–2.1 (additive), 2.1–2.4 (dominant), and >2.2 (recessive, for MAF 0.15, >3.5).

Genotype frequencies of all variants in cases were in HWE—rs1799750 (*p* = 0.083), rs486055 (*p* = 1.0), and rs2276109 (*p* = 0.369). There were no significant differences in genotype frequency between cases and controls (Table 2, Table 3 and Table 4).

### 3.1. Single-Locus Analysis—Case Group

The genotypic and allelic distributions of the three *MMP* polymorphisms (rs1799750, rs486055, and rs2276109) were analyzed in the context of their association with ACL injury-related outcomes. For rs1799750, the genotype frequencies were 30.2% for D/D, 50.9% for I/D, and 18.9% for I/I, with allele frequencies of 55.66% for D and 44.34% for I. Similarly, rs486055 showed genotype frequencies of 65.0% for C/C, 31.3% for C/T, and 3.8% for T/T, with allele frequencies of 80.6% for C and 19.4% for T. For rs2276109, the genotype frequencies were 80.0% for A/A, 17.5% for A/G, and 2.5% for G/G, with allele frequencies of 88.8% for A and 11.2% for G. The genotype distributions of all three SNPs were consistent with the Hardy–Weinberg equilibrium, i.e., rs1799750 (*p* = 0.749), rs486055 (*p* = 1.00), and rs2276109 (*p* = 0.115).

### 3.2. Association of MMP1 rs1799750, MMP10 rs486055, and MMP12 rs2276109 with ACLF, ACLS, ACLRP, and ACLRC

We assessed the association of the polymorphisms with various ACL-related conditions using several genetic models, namely codominant, dominant, recessive, overdominant.

*MMP1* rs1799750

ACLF (ACL injury frequency—single vs. multiple): Under the recessive model, the I/I genotype demonstrated a significantly higher odds of multiple ACL injuries compared to the combined D/D-I/D genotype (OR = 4.79, 95% CI: 2.05–11.19, *p* = 0.0002). The overdominant model revealed a protective effect for individuals with the I/D genotype compared to the combined D/D-I/I genotypes (OR = 0.49, 95% CI: 0.25–0.94, *p* = 0.031), as in Table 5.

ACLS (ACL strain): No significant associations were detected across all genetic models in the ACL strain cohort, as in Table 5.

ACLRP (ACL partial rupture): Significant findings were observed under the dominant model, where the combined I/D-I/I genotype presented a decreased risk compared to the D/D genotype (OR = 0.29, 95% CI: 0.14–0.62, *p* = 0.001. The overdominant model supported this observation, showing a reduced risk for the I/D genotype versus the combined D/D-I/I genotypes (OR = 0.33, 95% CI: 0.15–0.71, *p* = 0.0034), as in Table 5.

ACLRC (ACL complete rupture): The dominant model indicated a lower risk for the I/D-I/I genotype relative to the D/D genotype (OR = 0.26, 95% CI: 0.1–0.7, *p* = 0.0073)—Table 5.

*MMP10* rs486055

ACLF (ACL injury frequency—single vs. multiple): The analysis did not demonstrate significant associations in any genetic models. Notably, the recessive model indicated non-significant results with the T/T genotype and incremental allele increases, respectively, showing no significant influence on the frequency of ACL injuries, as in Table 6.

ACLS (ACL strain): No significant associations were observed across all genetic models for ACL strain, as in Table 6.

ACLRP (ACL partial rupture): Significant findings were observed in the codominant and dominant models for ACL partial rupture. Specifically, individuals carrying the C/T genotype showed a significantly increased risk compared to those with the C/C genotype (OR = 3.46, 95% CI: 1.62–7.38, *p* = 0.0048). The dominant model reported a similar increased risk for individuals carrying at least one T allele (OR = 3.09, 95% CI: 1.48–6.47, *p* = 0.0026). The overdominant model also highlighted an increased risk with the heterozygous genotype (OR = 3.47, 95% CI: 1.64–7.33, *p* = 0.0011), as in Table 6.

ACLRC (ACL complete rupture): In the ACL complete rupture cohort, no significant associations were found across any of the genetic models. This included the codominant, dominant, recessive, and overdominant, indicating no significant effect of the rs486055 polymorphism on the risk of complete ACL ruptures, as in Table 6.

*MMP12* rs2276109

ACLF (ACL injury frequency—single vs. multiple): Significant associations were observed in multiple models, although we have to interpret some of the data with caution when looking at 95% CIs, which are quite wide. In the codominant model (*p* = 0.004), the heterozygous A/G genotype was associated with an increased risk of multiple ACL injuries compared to the homozygous A/A genotype (OR = 3.53, 95% CI: 1.51–8.22). The homozygous G/G genotype further increased this risk (OR = 6.85, 95% CI: 0.69–67.89) but this effect was not significant. The dominant model combining A/G and G/G genotypes showed an even higher risk (OR = 3.8, 95% CI: 1.69–8.54, *p* = 0.0009). The overdominant model supported these significant associations, highlighting a strong influence of the G allele on ACL injury frequency, as in Table 7.

ACLS (ACL strain): No significant associations were found across any genetic models for ACL strain, indicating that the *MMP12* rs2276109 polymorphism may not influence this condition, as in Table 7.

ACLRP (ACL partial rupture): The codominant model did not show significant associations for partial ACL ruptures, with both A/G and G/G genotypes not differing significantly from the A/A genotype. Similarly, the dominant, recessive, and overdominant models showed no significant influence of the polymorphism on ACL partial ruptures, as in Table 7.

ACLRC (ACL complete rupture): Similar to ACLRP, no significant associations were observed in any models for ACL complete ruptures. This includes the codominant, dominant, recessive, and overdominant models, indicating no effect of the *MMP12* rs2276109 polymorphism on the risk of complete ACL ruptures, as in Table 7.

These findings highlight a specific and significant association of the *MMP12* rs2276109 polymorphism with the frequency of ACL injuries, particularly for individuals carrying one or more G alleles. In contrast, no significant associations were detected for ACL strain, partial rupture, or complete rupture, as in Table 7.

### 3.3. Multi-Locus Analysis

**Figure 1:**
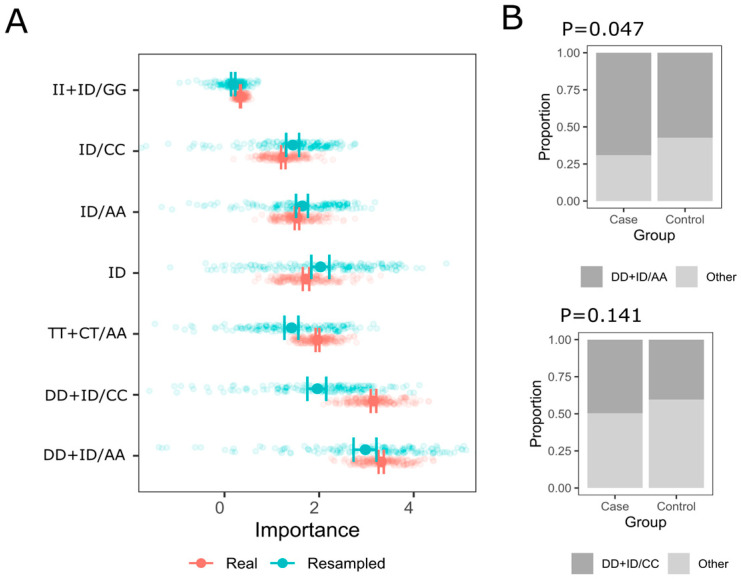
The most important interactions—*MMP1* rs1799750, *MMP10* rs486055, and *MMP12* rs2276109—selected by logic regression with mean values and 95% confidence intervals in the real and null (resampled) dataset for a case–control prediction. (**A**) Mean and 95% confidence intervals from the real and resampled (null) data set and 200 iterations (see Section 2) are shown; (**B**) bar plots showing a frequency distribution for the key genotype combinations in the case and control groups and Fisher’s exact test for comparison.

Multi-locus analysis was conducted using a logic regression method. Genotypes of *MMP1* rs1799750, *MMP10* rs486055, and *MMP12* rs2276109 were used as binary predictors of the case–control status. The most important combinations are presented in Figure 1. The highest importance metric was observed for the DD + ID/AA, DD + ID/CC, and TT + CT/AA compound genotypes. Notably, 95% confidence intervals did not overlap with those generated from the null dataset. The frequency distribution of the first two combinations is presented in Figure 1B, suggesting that DD + ID/AA is a high-risk combination (*p* = 0.047).

**Figure 2:**
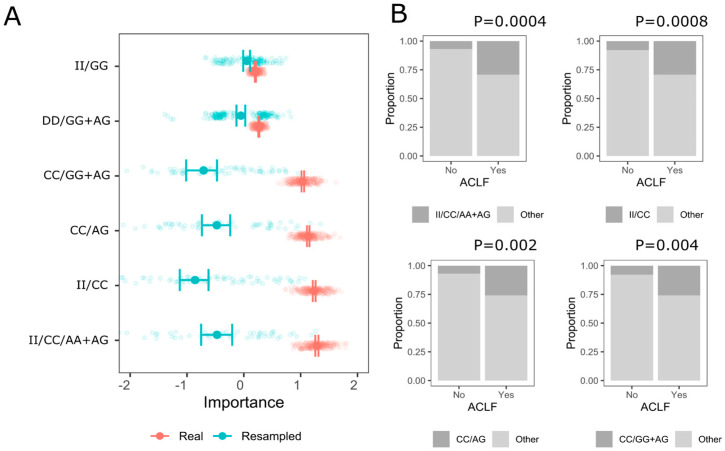
The most important interactions—*MMP1* rs1799750, *MMP10* rs486055, and *MMP12* rs2276109—selected by logic regression, with mean values and 95% confidence intervals in the real and null (resampled) dataset for the ACLF prediction. (**A**) Mean and 95% confidence intervals from the real and resampled (null) data set and 200 iterations (see Section 2) are shown; (**B**) bar plots showing a frequency distribution for the key genotype combinations in the multiple and single injury groups and Fisher’s exact test for comparison.

When the same approach was applied within the case group, only the ACLF phenotype could be predicted with compound genotypes (Figure 2A). The four combinations with the highest importance were II/CC/AA + AG, II/CC, CC/AG, and CC/GG + AG. Their 95% CIs were outside the null data set (95%CI) suggesting non-random selection of these interactions by logistic regression. The frequency distribution of these first four interactions differed significantly between ACLF groups (Figure 2B). For ACLS, ACLRP, and ACLRC we did not identify genotype combinations for which the importance metrics for real data significantly differed from the resampled (null) dataset. 

## 4. Discussion

This study investigated the association of three matrix metalloproteinase (MMP) polymorphisms—*MMP1* rs1799750, *MMP10* rs486055, and *MMP12* rs2276109—with the severity of various injuries of the anterior cruciate ligament. Utilizing various genetic models, we specifically explored their potential influence on ACL injury frequency and the grade of the injury from strains and partial ruptures to complete ruptures. We also evaluated the effect of inheriting various genotype combinations of these three MMP loci with injury severity. The findings highlighted distinct and context-specific associations for *MMP1* rs1799750 and *MMP12* rs2276109, while *MMP10* rs486055 exhibited more limited relevance.

### 4.1. MMP1 rs1799750

The analysis of *MMP1* rs1799750 revealed significant associations with multiple facets of ACL injuries. The recessive model highlighted a notably higher risk of recurrent ACL injuries for individuals with the I/I genotype compared to those inheriting the D/D or I/D genotypes. Conversely, the overdominant model indicated a reduced injury risk profile with the heterozygous I/D genotype, underscoring the complexity of genetics in modulating ACL injury risk.

Interestingly, while no significant associations were identified between *MMP1* rs1799750 and ACL strain, this polymorphism was linked to reduced risk of more severe injury phenotypes, such as either partial or complete ruptures, under several models. For partial ruptures, the dominant model implicated the I/D and I/I genotypes as having a reduced risk, consistent with findings from the overdominant model. These results collectively underscore the hypothesized contribution of *MMP1* rs1799750 as a genetic modulator of ACL injury severity and recurrence.

The broader relevance of MMP1 in ligament injury susceptibility is supported by prior studies emphasizing the central role of MMP1 in ECM remodeling, a key process in ligament function and repair [3,23,24]. Dysregulation of MMP1 activity, particularly through genetic polymorphisms, can impair ECM homeostasis, reducing ligament resilience and, therefore, influence injury risk. Similarly, Davis et al. [4] demonstrated that MMP1 activity, modulated by genetic and environmental factors, influences collagen turnover, inflammatory responses, and tissue remodeling in musculoskeletal injuries. This was also confirmed by Amar et al. [25], de Almeida, Luiz G.N. et al. [26], Lee and Kim [27], and Parks et al. [28]. The findings align with the well-documented role of MMPs in ECM remodeling, which is central to maintaining the structural integrity of ligaments and tendons. MMP1 and MMP12, in particular, have been implicated in collagen turnover and tissue repair processes, as highlighted by Davis et al. [4] and Kaynak et al. [29]. *MMP1* rs1799750, with its strong associations with ACL injury severity and recurrence, likely affects ECM integrity, contributing to ligament vulnerability or resilience depending on genotype. Similarly, Posthumus et al. [18] identified variants within *MMP1*, *MMP10*, and *MMP12* as key contributors to ligament susceptibility, reinforcing the centrality of these genes in ACL pathophysiology.

### 4.2. MMP10 rs486055

The *MMP10* rs486055 polymorphism demonstrated more limited associations with ACL-related injuries. While no significant findings were observed for ACL injury frequency, strain, or complete ruptures, the partial rupture profiles exhibited a notable exception. The codominant and dominant models implicated the T allele, particularly the C/T genotype, with an increased risk of sustaining a partial rupture. The overdominant model reinforced this observation, highlighting the heightened susceptibility of heterozygous individuals. However, due to the lack of significant associations across the other injury profiles, we hypothesize that *MMP10* rs486055 may play a more focused role in ACL pathophysiology, specifically in the context of partial ruptures.

These findings align with observations from Gibbon et al. [14] and Borzemska et al. [11], who noted variability in the contributions of ECM-related genes to distinct injury types. Ribbans et al. [3] further highlighted the context-specific nature of genetic associations, with certain polymorphisms, such as those in MMP10, potentially relevant only under specific mechanical or pathological conditions.

### 4.3. MMP12 rs2276109

The *MMP12* rs2276109 polymorphism showed significant associations with ACL injury frequency but not with the severity of the ACL-related injuries explored. The codominant and dominant models identified a substantial increase in the risk of multiple injuries among carriers of the G allele, with the heterozygous A/G and homozygous G/G genotypes exhibiting progressively increased odds ratios. These findings were further corroborated by the overdominant model, emphasizing the allele’s potential dose-dependent impact, which needs exploration. However, no significant associations emerged for ACL strain, partial rupture, or complete rupture, suggesting that the effect of *MMP12* rs2276109 is likely restricted to injury recurrence rather than severity.

This finding resonates with such studies as Feldmann et al. [30] and Malila et al. [12], which highlighted the role of ECM-related genetic pathways, including those involving MMPs, in injury susceptibility and recurrence. Feldmann et al. [30] also emphasized the importance of considering population-specific genetic variability, which may explain the differences in the significance of MMP12 variants across studies.

### 4.4. Biological Mechanisms and Functional Implications

The findings align with the well-documented role of MMPs in ECM remodeling, which is central to maintaining the structural integrity of ligaments and tendons. MMP1 and MMP12, in particular, have been implicated in collagen turnover and tissue repair processes, as highlighted by Davis et al. [4]. MMP1 rs1799750, with its strong associations with ACL injury severity and recurrence, likely affects ECM integrity, contributing to ligament vulnerability or resilience depending on genotype. Similarly, Posthumus et al. [18] identified MMP1, MMP10, and MMP12 as key contributors to ligament susceptibility, reinforcing the centrality of these genes in ACL pathophysiology.

### 4.5. Comparative Insights with Other Soft Tissue Injuries

The role of MMPs in ligament injuries is consistent with their involvement in other musculoskeletal conditions, such as Achilles tendinopathy. Gibbon et al. [14] demonstrated the association of MMP variants with soft tissue injuries, suggesting that dysregulated ECM turnover is a shared mechanism across injury types. Borzemska et al. [11] expanded on this by discussing how genetic predispositions, such as MMP polymorphisms, interact with external factors to heighten the risk of non-contact injuries in tendons and ligaments. Similar associations between *MMP* variants and soft tissue injuries have been reported in studies on Achilles tendinopathy and rotator cuff tears, supporting the role of ECM remodeling genes in musculoskeletal injury susceptibility [31,32,33,34,35]. Associations between *MMP* variants and soft tissue pathologies have been also reported in carpal tunnel syndrome by Burger et al. [17] and in lateral epicondylitis by Riley [36]. Moreover, *MMP* gene variants have been linked to patellar tendinopathy [37], highlighting the broad contribution of ECM remodeling enzymes to tendon and ligament pathology across multiple anatomical sites.

### 4.6. Environmental and Biomechanical Interactions

The interaction between genetic predisposition and environmental stressors is crucial for understanding ACL injury risk. Meeuwisse et al. [19] introduced a dynamic model of etiology in sports injuries, illustrating how cumulative factors, including genetics and biomechanical loads, interact to exceed an injury threshold. Ribbans et al. [3] further developed this concept with the “Jar Model”, explaining how genetic risk factors, like MMP1 rs1799750, amplify the impact of environmental stressors, such as high-intensity sports, to increase injury susceptibility.

### 4.7. Epigenetics and Gene Regulation

Epigenetic modifications provide an additional layer of complexity in regulating MMP activity. Vigetti et al. [38] and Gomez et al. [39] highlighted how DNA methylation and histone acetylation influence ECM gene expression, potentially modulating individual responses to mechanical stress or injury. Such regulatory mechanisms may explain why individuals with similar genotypes exhibit varying injury outcomes, underscoring the importance of integrating epigenetic profiling in future studies.

### 4.8. Clinical and Translational Implications

The identification of genetic markers, such as MMP1 rs1799750 and MMP12 rs2276109, offers significant potential for personalized medicine. Polygenic risk scores combining these polymorphisms with other ECM-related variants (e.g., TNC, COL1A1) could enhance the predictive accuracy for ACL injuries [13]. Gumucio et al. [40] further highlighted the therapeutic potential of targeting MMP activity, such as with localized inhibitors or platelet-rich plasma (PRP) therapies, to optimize ligament healing and prevent reinjury.

### 4.9. Methodological Considerations and Future Directions

Although this study offers important perspectives, several limitations must be addressed. The modest sample size may have limited the detection of subtle genetic effects, as noted by Ioannidis et al. [41]. We did not pre-specify sample size, but post hoc analysis indicated a limited power for small recessive effects at low MAF. Exploring gene–gene interactions, as demonstrated by Feldmann et al. [30] and Dlamini et al. [42] could reveal synergistic effects that remain undetected in single-gene analyses.

Future research should prioritize longitudinal studies to track how genetic predispositions influence injury risk and recovery over time. Advanced genomic approaches, such as whole exome sequencing and whole genome sequencing, could uncover additional variants and regulatory mechanisms linked to ligament biology [29,30,31]. Moreover, integrating genetic, epigenetic, and proteomic data could clarify genotype–phenotype relationships and refine predictive models for injury prevention [43].

## 5. Conclusions

This study highlights the complex role of *MMP* polymorphisms in ACL injury susceptibility, severity, and recurrence. In conclusion, *MMP1* rs1799750 showed a protective effect against ACL injury, *MMP10* rs486055 was associated with an increased risk of partial rupture, and *MMP12* rs2276109 was linked to higher injury frequency. These results emphasize the role of genetic variants in modulating ligament resilience and recurrence risk. By integrating genetic findings with biomechanical, environmental, and epigenetic data, future research can advance predictive models and targeted interventions. These efforts hold the potential to transform injury prevention and management, paving the way for personalized approaches in high-risk populations.

## Figures and Tables

**Table 1:** Group characteristics.

Characteristic	Controls, N = 136 ^1^	Cases, N = 160 ^1^	*p* ^2^
Age (years)	27 (4)	36 (7)	<0.001
Height (cm)	178 (7)	178 (6)	0.284
Missing	0	1	
Body mass (kg)	73 (16)	78 (10)	<0.001
Missing	0	1	

What ^1^ mean (SD), ^2^ Wilcoxon rank sum test.

**Table 2:** The allele frequency distributions for rs1799750 between cases and controls.

Model	Cases N (%)		Controls N (%)		OR (95% CI)			P/FDR P
Codominant								0.018/0.072
D/D	34 (25.0)		48 (30.4)		1.00			
I/D	57 (41.9)		80 (50.6)		1.70 (0.80; 3.62)			
I/I	45 (33.1)		30 (19.0)		0.59 (0.26; 1.35)			
Dominant								0.728/0.728
D/D	34 (25.0)		48 (30.4)		1.00			
I/D-I/I	102 (75.0)		110 (69.6)		1.13 (0.57; 2.23)			
Recessive								0.014/0.072
D/D-I/D	91 (66.9)		128 (81.0)		1.00			
I/I	45 (33.1)		30 (19.0)		0.42 (0.21; 0.85)			
Overdominant								0.011/0.072
D/D-I/I	79 (58.1)		78 (49.4)		1.00			
I/D	57 (41.9)		80 (50.6)		2.23 (1.18; 4.19)			

**Table 3:** The allele frequency distributions for rs486055 between cases and controls.

Model	Cases N (%)		Controls N (%)		OR (95% CI)			*p*
Codominant								0.674/0.728
C/C	98 (72.1)		103 (64.8)		1.00			
C/T	35 (25.7)		50 (31.4)		1.21 (0.61; 2.41)			
T/T	3 (2.2)		6 (3.8)		0.54 (0.08; 3.55)			
Dominant								0.718/0.728
C/C	98 (72.1)		103 (64.8)		1.00			
C/T-T/T	38 (27.9)		56 (35.2)		1.13 (0.58; 2.19)			
Recessive								0.486/0.716
C/C-C/T	133 (97.8)		153 (96.2)		1.00			
T/T	3 (2.2)		6 (3.8)		0.51 (0.08; 3.33)			
Overdominant								0.537/0.716
C/C-T/T	101 (74.3)		109 (68.6)		1.00			
C/T	35 (25.7)		50 (31.4)		1.24 (0.63; 2.45)			

**Table 4:** The allele frequency distributions for rs2276109 between cases and controls.

Model	Cases N (%)		Controls N (%)		OR (95% CI)			*p*
Codominant								0.209/0.418
A/A	106 (77.9)		128 (80.5)		1.00			
A/G	30 (22.1)		27 (17.0)		1.50 (0.70; 3.20)			
G/G	0 (0.0)		4 (2.5)		0.00			
Dominant								0.206/0.418
A/A	106 (77.9)		128 (80.5)		1.00			
A/G-G/G	30 (22.1)		31 (19.5)		1.62 (0.77; 3.41)			
Recessive								0.152/0.418
A/A-A/G	136 (100.0)		155 (97.5)		1.00			
G/G	0 (0.0)		4 (2.5)		0.00			
Overdominant								0.324/0.555
A/A-G/G	106 (77.9)		132 (83.0)		1.00			
A/G	30 (22.1)		27 (17.0)		1.46 (0.68; 3.13)			

**Table 5:** Association of *MMP1* rs1799750 with ACLF, ACLS, ACLRP, and ACLRC.

Outcome	Model	Genotype	0N (%)	1 N (%)	OR (95% CI)	P/FDR P
ACLF (0—single, 1—multiple)	Cod	D/D	33 (32.7)	15 (25.9)	1.00	0.001/0.0096
		I/D	58 (57.4)	23 (39.7)	0.87 (0.40; 1.90)	
		I/I	10 (9.9)	20 (34.5)	4.40 (1.66; 11.65)	
	Dom	D/D	33 (32.7)	15 (25.9)	1.00	0.365/0.631
		I/D-I/I	68 (67.3)	43 (74.1)	1.39 (0.68; 2.86)	
	Rec	D/D-I/D	91 (90.1)	38 (65.5)	1.00	0.0002/0.0096
		I/I	10 (9.9)	20 (34.5)	4.79 (2.05; 11.19)	
	Over	D/D-I/I	43 (42.6)	35 (60.3)	1.00	0.031/0.099
		I/D	58 (57.4)	23 (39.7)	0.49 (0.25; 0.94)	
ACLS (0—no, 1—yes)	Cod	D/D	12 (38.7)	36 (28.1)	1.00	0.468/0.702
		I/D	13 (41.9)	68 (53.1)	1.74 (0.72; 4.21)	
		I/I	6 (19.4)	24 (18.8)	1.33 (0.44; 4.04)	
	Dom	D/D	12 (38.7)	36 (28.1)	1.00	0.257/0.549
		I/D-I/I	19 (61.3)	92 (71.9)	1.61 (0.71; 3.66)	
	Rec	D/D-I/D	25 (80.6)	104 (81.2)	1.00	0.939/1.0
		I/I	6 (19.4)	24 (18.8)	0.96 (0.36; 2.60)	
	Over	D/D-I/I	18 (58.1)	60 (46.9)	1.00	0.263/0.549
		I/D	13 (41.9)	68 (53.1)	1.57 (0.71; 3.47)	
ACLRP (0—no, 1—yes)	Cod	D/D	28 (23.3)	20 (51.3)	1.00	0.003/0.018
		I/D	69 (57.5)	12 (30.8)	0.24 (0.11; 0.56)	
		I/I	23 (19.2)	7 (17.9)	0.43 (0.15; 1.18)	
	Dom	D/D	28 (23.3)	20 (51.3)	1.00	0.001/0.0096
		I/D-I/I	92 (76.7)	19 (48.7)	0.29 (0.14; 0.62)	
	Rec	D/D-I/D	97 (80.8)	32 (82.1)	1.00	0.865/0.944
		I/I	23 (19.2)	7 (17.9)	0.92 (0.36; 2.35)	
	Over	D/D-I/I	51 (42.5)	27 (69.2)	1.00	0.003/0.018
		I/D	69 (57.5)	12 (30.8)	0.33 (0.15; 0.71)	
ACLRC (0—no, 1—yes)	Cod	D/D	37 (26.4)	11 (57.9)	1.00	0.006/0.024
		I/D	73 (52.1)	8 (42.1)	0.37 (0.14; 0.99)	
		I/I	30 (21.4)	0 (0.0)	0.00 (0.00)	
	Dom	D/D	37 (26.4)	11 (57.9)	1.00	0.007/0.026
		I/D-I/I	103 (73.6)	8 (42.1)	0.26 (0.10; 0.70)	
	Rec	D/D-I/D	110 (78.6)	19 (100.0)	1.00	0.025/0.086
		I/I	30 (21.4)	0 (0.0)	0.00 (0.00)	
	Over	D/D-I/I	67 (47.9)	11 (57.9)	1.00	0.411/0.680
		I/D	73 (52.1)	8 (42.1)	0.67 (0.25; 1.76)	

ACLF—ACL injury frequency, ACLS—ACL strain, ACLRP—ACL partial rupture, ACLRC—ACL complete rupture, Cod—codominant model, Dom—dominant model, Rec—recessive model, and Over—overdominant model.

**Table 6:** Association of *MMP10* rs486055 with ACLF, ACLS, ACLRP, and ACLRC.

Outcome	Model	Genotype	0N (%)	1 N (%)	OR (95% CI)	P
ACLF (0—single, 1—multiple)	Cod	C/C	62 (61.4)	42 (71.2)	1.00	0.444/0.687
		C/T	35 (34.7)	15 (25.4)	0.63 (0.31; 1.30)	
		T/T	4 (4.0)	2 (3.4)	0.74 (0.13; 4.21)	
	Dom	C/C	62 (61.4)	42 (71.2)	1.00	0.207/0.549
		C/T-T/T	39 (38.6)	17 (28.8)	0.64 (0.32; 1.28)	
	Rec	C/C-C/T	97 (96.0)	57 (96.6)	1.00	0.854/0.944
		T/T	4 (4.0)	2 (3.4)	0.85 (0.15; 4.79)	
	Over	C/C-T/T	66 (65.3)	44 (74.6)	1.00	0.220/0.549
		C/T	35 (34.7)	15 (25.4)	0.64 (0.31; 1.31)	
ACLS (0—no, 1—yes)	Cod	C/C	19 (61.3)	85 (65.9)	1.00	0.850/0.944
		C/T	11 (35.5)	39 (30.2)	0.79 (0.34; 1.82)	
		T/T	1 (3.2)	5 (3.9)	1.12 (0.12; 10.13)	
	Dom	C/C	19 (61.3)	85 (65.9)	1.00	0.632/0.798
		C/T-T/T	12 (38.7)	44 (34.1)	0.82 (0.36; 1.84)	
	Rec	C/C-C/T	30 (96.8)	124 (96.1)	1.00	0.862/0.944
		T/T	1 (3.2)	5 (3.9)	1.21 (0.14; 10.74)	
	Over	C/C-T/T	20 (64.5)	90 (69.8)	1.00	0.574/0.798
		C/T	11 (35.5)	39 (30.2)	0.79 (0.34; 1.80)	
ACLRP (0—no, 1—yes)	Cod	C/C	86 (71.7)	18 (45.0)	1.00	0.005/0.022
		C/T	29 (24.2)	21 (52.5)	3.46 (1.62; 7.38)	
		T/T	5 (4.2)	1 (2.5)	0.96 (0.11; 8.68)	
	Dom	C/C	86 (71.7)	18 (45.0)	1.00	0.003/0.018
		C/T-T/T	34 (28.3)	22 (55.0)	3.09 (1.48; 6.47)	
	Rec	C/C-C/T	115 (95.8)	39 (97.5)	1.00	0.616/0.798
		T/T	5 (4.2)	1 (2.5)	0.59 (0.07; 5.20)	
	Over	C/C-T/T	91 (75.8)	19 (47.5)	1.00	0.001/0.0096
		C/T	29 (24.2)	21 (52.5)	3.47 (1.64; 7.33)	
ACLRC (0—no, 1—yes)	Cod	C/C	94 (66.7)	10 (52.6)	1.00	0.496/0.721
		C/T	42 (29.8)	8 (42.1)	1.79 (0.66; 4.86)	
		T/T	5 (3.5)	1 (5.3)	1.88 (0.20; 17.73)	
	Dom	C/C	94 (66.7)	10 (52.6)	1.00	0.237/0.549
		C/T-T/T	47 (33.3)	9 (47.4)	1.80 (0.68; 4.73)	
	Rec	C/C-C/T	136 (96.5)	18 (94.7)	1.00	0.725/0.870
		T/T	5 (3.5)	1 (5.3)	1.51 (0.17; 13.67)	
	Over	C/C-T/T	99 (70.2)	11 (57.9)	1.00	0.287/0.574
		C/T	42 (29.8)	8 (42.1)	1.71 (0.64; 4.57)	

ACLF—ACL injury frequency, ACLS—ACL strain, ACLRP—ACL partial rupture, ACLRC—ACL complete rupture, Cod—codominant model, Dom—dominant model, Rec—recessive model, and Over—overdominant model.

**Table 7:** Association of *MMP12* rs2276109 with ACLF, ACLS, ACLRP, and ACLRC.

Outcome	Model	Genotype	0N (%)	1 N (%)	OR (95% CI)	P
ACLF (0—single, 1—multiple)	Cod	A/A	89 (88.1)	39 (66.1)	1.00	0.004/0.021
		A/G	11 (10.9)	17 (28.8)	3.53 (1.51; 8.22)	
		G/G	1 (1.0)	3 (5.1)	6.85 (0.69; 67.89)	
	Dom	A/A	89 (88.1)	39 (66.1)	1.00	0.0009/0.0096
		A/G-G/G	12 (11.9)	20 (33.9)	3.80 (1.69; 8.54)	
	Rec	A/A-A/G	100 (99.0)	56 (94.9)	1.00	0.116/0.348
		G/G	1 (1.0)	3 (5.1)	5.36 (0.54; 52.73)	
	Over	A/A-G/G	90 (89.1)	42 (71.2)	1.00	0.005/0.022
		A/G	11 (10.9)	17 (28.8)	3.31 (1.43; 7.69)	
ACLS (0—no, 1—yes)	Cod	A/A	27 (87.1)	101 (78.3)	1.00	0.598/0.798
		A/G	4 (12.9)	24 (18.6)	1.60 (0.51; 5.02)	
		G/G	0 (0.0)	4 (3.1)	0.00	
	Dom	A/A	27 (87.1)	101 (78.3)	1.00	0.252/0.549
		A/G-G/G	4 (12.9)	28 (21.7)	1.87 (0.60; 5.80)	
	Rec	A/A-A/G	31 (100.0)	125 (96.9)	1.00	1.0/1.0
		G/G	0 (0.0)	4 (3.1)	0.00	
	Over	A/A-G/G	27 (87.1)	105 (81.4)	1.00	0.440/0.687
		A/G	4 (12.9)	24 (18.6)	1.54 (0.49; 4.82)	
ACLRP (0—no, 1—yes)	Cod	A/A	94 (78.3)	34 (85.0)	1.00	0.611/0.798
		A/G	23 (19.2)	5 (12.5)	0.60 (0.21; 1.71)	
		G/G	3 (2.5)	1 (2.5)	0.92 (0.09; 9.16)	
	Dom	A/A	94 (78.3)	34 (85.0)	1.00	0.340/0.628
		A/G-G/G	26 (21.7)	6 (15.0)	0.64 (0.24; 1.68)	
	Rec	A/A-A/G	117 (97.5)	39 (97.5)	1.00	1.0/1.0
		G/G	3 (2.5)	1 (2.5)	1.00 (0.10; 9.89)	
	Over	A/A-G/G	97 (80.8)	35 (87.5)	1.00	0.322/0.618
		A/G	23 (19.2)	5 (12.5)	0.60 (0.21; 1.71)	
ACLRC (0—no, 1—yes)	Cod	A/A	111 (78.7)	17 (89.5)	1.00	0.718/0.870
		A/G	26 (18.4)	2 (10.5)	0.50 (0.11; 2.31)	
		G/G	4 (2.8)	0 (0.0)	0.00 (0.00)	
	Dom	A/A	111 (78.7)	17 (89.5)	1.00	0.240/0.549
		A/G-G/G	30 (21.3)	2 (10.5)	0.44 (0.10; 1.99)	
	Rec	A/A-A/G	137 (97.2)	19 (100.0)	1.00	1.0/1.0
		G/G	4 (2.8)	0 (0.0)	0.00 (0.00)	
	Over	A/A-G/G	115 (81.6)	17 (89.5)	1.00	0.368/0.631
		A/G	26 (18.4)	2 (10.5)	0.52 (0.11; 2.39)	

ACLF—ACL injury frequency, ACLS—ACL strain, ACLRP—ACL partial rupture, ACLRC—ACL complete rupture, Cod—codominant model, Dom—dominant model, Rec—recessive model, and Over—overdominant model.

## Data Availability

The data presented in this study are available on request from the corresponding author. The data are not publicly available due to ethical restrictions involving human participant privacy.
